# Post-Synthetic Defucosylation of AGP by *Aspergillus nidulans* α-1,2-Fucosidase Expressed in *Arabidopsis* Apoplast Induces Compensatory Upregulation of α-1,2-Fucosyltransferases

**DOI:** 10.1371/journal.pone.0159757

**Published:** 2016-07-22

**Authors:** Gennady V. Pogorelko, Nathan T. Reem, Zachary T. Young, Lauran Chambers, Olga A. Zabotina

**Affiliations:** 1 Roy J Carver Department of Biochemistry, Biophysics and Molecular Biology, Iowa State University, 3212 MMB, Ames, IA, United States of America; 2 Department of Plant Pathology and Microbiology, Iowa State University, 219 Bessey Hall, Ames, IA, United States of America; Institute for Sustainable Plant Protection, C.N.R., ITALY

## Abstract

Cell walls are essential components of plant cells which perform a variety of important functions for the different cell types, tissues and organs of a plant. Besides mechanical function providing cell shape, cell walls participate in intercellular communication, defense during plant-microbe interactions, and plant growth. The plant cell wall consists predominantly of polysaccharides with the addition of structural glycoproteins, phenolic esters, minerals, lignin, and associated enzymes. Alterations in the cell wall composition created through either changes in biosynthesis of specific constituents or their post-synthetic modifications in the apoplast compromise cell wall integrity and frequently induce plant compensatory responses as a result of these alterations. Here we report that post-synthetic removal of fucose residues specifically from arabinogalactan proteins in the Arabidopsis plant cell wall induces differential expression of fucosyltransferases and leads to the root and hypocotyl elongation changes. These results demonstrate that the post-synthetic modification of cell wall components presents a valuable approach to investigate the potential signaling pathways induced during plant responses to such modifications that usually occur during plant development and stress responses.

## Introduction

The cell wall is one of the major plant cell compartments participating in multiple important processes. Cell walls serve as a mechanical barrier to protect cells against environmental cues, determine cell shape, provide turgor pressure feedback, and support the whole plants’ stature. Recently, involvement of the cell wall in signaling mechanisms during plant growth and response to biotic and abiotic stresses was proposed, and the term Cell Wall Integrity (CWI) control was introduced [1–4].

Plant cell walls are mainly composed of different types of polysaccharides that can be arbitrarily divided into three major groups: cellulose, hemicelluloses, and pectin [5]. The hemicelluloses include β-(1–3,1–4)-glucan, xyloglucan, xylans, and mannans. Pectin is a complex polysaccharide structure that is sub-divided into homogalacturonan, xylogalacturonan, apiogalacturonan, rhamnogalacturonan-I (RG-I), and rhamnogalacturonan-II (RG-II) [5–6]. The cell wall also contains a small amount of various glycoproteins, and in some specialized tissues, lignin. In addition, there are a large number of cell wall modifying enzymes and structural glycoproteins localized in the cell wall. Among the most common cell wall localized glycoproteins are arabinogalactan proteins (AGP), hydroxyproline-rich glycoproteins (HRGP), proline-rich proteins (PRP) and glycine-rich proteins (GRP).

Although the sugar composition of polysaccharides varies between different plant species and even tissues in the same plant [7], there are some compositional features which determine the specific types of polysaccharide. Thus, there are three polysaccharides that contain l-fucose in their structure. Xyloglucan is generally composed of β-1,4-linked d-glucose backbone branched with monomeric d-xylose, xylose-galactose, xylose-arabinose or xylose-xylose disaccharides. In some plants including Arabidopsis, xyloglucan also contains trisaccharide branches composed of xylose, galactose and terminal fucose [8]. In pectin, both RG-I and RG-II contain fucose in their structures, but with different types of linkages [6, 9]. The glycans in AGP are primarily O-linked to the Hyp residues of the protein backbone and generally composed of (1–3)-β-d-galactoses with side chains terminated by (1–3)-/(1–4)-d-arabinose, (1–6)-d-glucuronic acid, (1–3)-/(1–4)-l-rhamnose, or (1–2)-l-fucose, where fucose is bound to arabinose [10–11].

The gene family GT37 contains the genes proposed to encode fucosyltransferases (FUTs), the enzymes which add d-fucose residues to various cell wall polymers, including xyloglucan, RG-I, RG-II, and AGP [10, 12–16]. Arabidopsis has ten FUTs belonging to the GT37 family and are predicted to be involved in cell wall biosynthesis [14, 17]. The works of Perrin et al. [12, 18] and Vanzin et al. [15] provided strong evidence that FUT1 is a single FUT protein responsible for fucosylation of xyloglucan in Arabidopsis. In addition, Arabidopsis FUT4 and FUT6 were demonstrated to fucosylate AGPs [10, 19–20]. No information about functional activity of the other seven FUT proteins are currently available, except that AtFUT2, AtFUT9, and AtFUT10 have extremely low expression levels, and that AtFUT10 is mainly expressed in Arabidopsis stem tissues [14]. Also, there is no information about which of the Arabidopsis FUT proteins add fucoses to the RG-I and RG-II polysaccharides.

Although fucose residues are a comparatively minor component in different cell wall polysaccharides and glycoproteins, the presence of this monosaccharide can dramatically affect structural organization of cell wall polysaccharides and their function. For example, it was shown that the presence of terminal fucose in xyloglucan polysaccharides is important in regulating cell wall integrity and extensibility [10], and the highest expression of *AtFUT1* was detected in the elongating inflorescence stems of Arabidopsis [18]. However, it was recently reported that increased degree of xyloglucan fucosylation does not affect the growth and development of Arabidopsis plants [21]. In addition, the fucosylated xyloglucan fragments were shown to be involved in regulation of expansion by acting as signal molecules [22].

AGP is another important component of the plant cell wall, and it was proposed that fucosylation of these molecules is required for normal root cell elongation [16]. However more recently it was shown that the roots of Arabidopsis double mutant plants *fut4fut6*, whose cell wall AGPs completely lack fucosyl residues, had the same length as wild type plants in normal conditions, but showed increased susceptibility to high salt stress [19–20]. On the other hand, constitutive expression of a human Lewis fucosyltransferase (hFUT3) in tobacco plants, involved in glycoprotein fucosylation, resulted in delayed growth and shorter roots [23].

A valuable approach to investigate the impact of cell wall compositional alterations on plant phenotype is the constitutive overexpression of specific cell wall modifying proteins in the apoplast, which cause specific cell wall modifications post-synthetically. For example, it was shown that expression of the microbial polygalacturonase in Arabidopsis or Tobacco apoplast decreased homogalacturonan content in the cell wall and reduced plant growth, but increased their resistance to fungal infection [24]. Reduction of polysaccharide acetylation by expression of microbial acetyl esterases in Arabidopsis and Brachypodium increased plant resistance to fungal infection without impacting plant growth and development [25]. Transgenic Arabidopsis plants expressing a pectin methylesterase inhibitors (AtPMEI-1 and AtPMEI-2), that increase pectin esterification in cell wall, grew larger in comparison with wild type plants [26]. Cell wall modifications introduced post-synthetically can have different impacts on plant growth or stress response in comparison with alterations caused by knocking out of biosynthetic enzymes.

In this study, *A*. *nidulans* α-1,2-fucosidase was expressed in Arabidopsis apoplast and demonstrated to cleave fucosyl residues primarily from cell wall AGP molecules. This post-synthetic defucosylation of AGP glycan affects the growth of Arabidopsis seedlings and induces differential expression of FUT genes in an organ-specific manner, most likely as a compensatory plant response.

## Materials and Methods

### Construction of transformation vectors and transformation of Arabidopsis

The *A*. *nidulans* α-fucosidase (AN8149.2) (*AnF*) gene was amplified by Platinum *Pfx* DNA-polymerase (Invitrogen, 11708–013) from plasmids extracted from *Pichia pastoris* strains harboring these genes [27] obtained from The Fungal Genetics Stock Center (www.fgsc.net) using appropriate Forward (containing *Xba*I restriction enzyme site) and Reverse (containing *Kpn*I restriction enzyme site) primers and after T-tailing cloned into the pGEM-T Easy vector (Promega, A1360) (all primer sequences are shown in S1 Table). Using *Kpn*I/*Xba*I restriction enzymes, the AnF gene was moved into pENTR D-TOPO vector (Invitrogen, K2400-20) linearized with the same enzymes and containing cloned coding sequence of the *A*. *thaliana* β-expansin signal peptide. The signal peptide was amplified from pBAt plasmid (obtained from Dr. Hahn, CCRC) using AtSP-F and AtSP-R (containing *Kpn*I and *Xba*I restriction enzyme sites) primers. Using Gateway technology and Gateway LR Clonase II enzyme mix (Invitrogen, 11791–020), fragments containing the β-expansin signal peptide fused with the *Aspergillus* gene were moved into the pEarleyGate101 binary vector obtained from ABRC (http://abrc.osu.edu). Obtained binary vector was transformed into *Agrobacterium tumefaciens* strain GV3101 cells by electroporation, and *A*. *thaliana* plants were transformed with these cells by the floral-dip method [28].

### Screening of transformed plants and confocal microscopy

Transgenic plants were selected by spraying 5-day old *A*. *thaliana* seedlings (cotyledon stage) with herbicide Rely 2000 (Glufosinate-ammonium, 250 mg/L) once every 2 days 5–6 times. T2 generation of herbicide resistant plants were confirmed by PCR and observed under confocal microscope.

For microscopy analysis plants were grown in a controlled growth chamber under normal day conditions (16 h light/8 h dark) at 170–180 μmol m^−2^ s^−1^ light intensity and 21°C. Localization of AnF-YFP-fusion protein in the cell wall was examined using a Leica SP5 X confocal microscope; YFP was examined using 488 nm excitation and 500 to 570 nm emission wavelengths.

### Apoplastic fluid preparation

Aerial parts (stems, leaves, siliques, flowers, and buds) of 3-week old individual plants were individually harvested and immediately cut into 5 mm segments using a sharp razor blade, then placed vertically into a 10 ml syringe which had its tip sealed with Parafilm. Five ml of pre-cooled extraction buffer (25 mM Tris-HCl, 50mM EDTA, 150 mM MgCl_2_, pH 7.4) was added and the syringe was placed under vacuum twice for 30 minutes with a 5 minute break. After vacuum infiltration, the buffer was carefully drained and the syringe was placed into a centrifuge tube and centrifuged at 4000g for 10 min. Apoplastic fluid, which accumulated at the bottom of the tube, was collected into a new vial and kept on ice until assayed. All the above steps were performed in the cold room at 4°C. Protein concentration was determined using Bradford assay [29]. For Western analysis, total protein was precipitated from apoplastic extract by addition of trichloroacetic acid (TCA) to a final concentration of 10% (v/v). Proteins were pelleted by centrifugation at 8000g for 20 min, washed with acetone and air-dried.

### Western blot analysis of apoplastic proteins

Apoplastic proteins were re-suspended in 1X Laemmli’s buffer (125mM Tris-HCl (pH 6.8); 1% SDS; 37.7% glycerol; 1% β-mercaptoethanol; 0.01% bromophenol blue), boiled for 5 minutes and used immediately for SDS-PAGE. Fifteen micrograms of total protein were loaded per well for 10% SDS-PAGE. After separation, the proteins were electrophoretically transferred to a Trans-Blot Transfer Medium membrane (Bio-Rad, 162–0112) using the Trans-Blot Cell (Bio-Rad) with a standard transfer buffer. Primary Anti-GFP monoclonal antibody (Covance, CA, USA, MMS-118P) at a dilution of 1:5000 and secondary Anti-Mouse IgG (whole molecule)-Peroxidase antibody (Sigma, A9044) at a dilution of 1:40000 were used for detection of YFP-fusion proteins. Membranes were treated with the HyGlo Quick Spray Reagent B for peroxidase activity (Denville, E2400) and immediately visualized by ChemiDoc™ XRS+ System (BioRad, 170–8265).

### Assay for α-fucosidase enzymatic activity

For α-fucosidase activity assay, 1 mL of reaction mixture containing 500 μg of apoplast proteins and 0.2 mM 2-fucosyllactose (Carbosynth) in 50 mM ammonium formate buffer, pH 4.5, was incubated at 28°C and aliquots were taken for different time points. Released fucose was quantified using a high performance anion-exchange chromatography with pulsed amperometric detection (HPAEC–PAD) (Dionex, Sunnyvale, CA) on a CarboPac PA20 column with post-column addition of 300mM NaOH, using following gradient conditions: 0–0.05 min, 12mM NaOH; 0.05–26 min, 0.65mM NaOH; 26.1–46 min, 1–300mM NaOH; 46.1–55 min, and 12mM NaOH [30]. Amount of released fucose was estimated using a standard curve made by analyzing standard fucose solutions of different concentrations using the same gradient conditions.

### Analysis of cell wall composition

Cell walls were prepared from the plant tissues remaining after apoplastic fluid extraction as described previously [31]. Briefly, alcohol insoluble residue extraction was done as follows. 1–5 grams of tissue was ground with a mortar and pestle in liquid nitrogen and suspended in 13 mL of 80% ethanol followed by incubation in an 80°C water bath for 1 hour. Samples were then treated with Polytron at 25,000 rpm until pieces were well homogenized. After a 10 minute centrifugation at 13,000g, the pellet was re-suspended in 13 mL of 80% ethanol and again incubated for 1 hour at 80°C, followed by 3 washes with 10 mL of fresh 80% ethanol and one wash of 10 mL 80% acetone. The pellet was suspended in 15 mL 0.5% SDS + 5 mM Na_2_S_2_O_5_ and mixed on a shaker at 4°C overnight and washed 5 times with 10 ml of dH_2_O prior to a 20 minute incubation in 10 mL of 1:1 (v/v) chloroform: methanol. After one wash with 100% acetone, the pellet was dried in a 50°C oven until completely dry.

Starch was removed from samples heated in advance in 0.1 M Potassium Phosphate buffer (pH 5.8) + 0.02% thimerosal for 20 min in an 80°C water bath, followed by addition of amylase (100 U/mL solution) and incubated for 48 hours at 37°C. The remaining pellet was then washed 5 times with dH_2_O and dried in a 50°C oven.

Pectic material was extracted by incubation of cell wall material in 10 mL of 50 mM CDTA + 0.02% Thimerosal (pH 7.5) overnight at room temperature.

Isolation of hemicelluloses was done in 2 steps with 1M KOH and 4M KOH. At the first step the treatment was performed in 10 mL of 1M KOH + 0.1% NaBH_4_ on shaker overnight at room temperature. The insoluble fraction was separated by centrifugation at 10,000g and supernatant was neutralized using glacial acetic acid followed by dialysis against four changes of 4 L of deionized water and then lyophilized. The second step of pellet treatment using 4M KOH was performed similarly and the amount of glacial acetic acid for neutralization was increased accordingly to keep a 1:1 molar ratio according to the KOH concentration.

Xyloglucan was characterized using XEG digestion and quantification of subunits by HPAEC as described earlier by Zabotina et al. [31].

Isolation of AGP was performed according to the protocol described by Schultz et al [32]. Briefly, 10 g of leaf tissue (fresh weight) was ground in liquid nitrogen and was added to 10 mL of extraction buffer (50 mM Tris-HCl, pH 8.0, 10 mM EDTA, 0.1% β-mercaptoethanol, and 1% (w/v) Triton X-100) and incubated at 4°C for 3 hr. After centrifugation for 10 min at 14,000g, the supernatant was precipitated with 5 volumes of ethanol at 4°C for 16 hours. The pellet was resuspended in 5 mL of 50 mM Tris-HCl (pH 8.0) and the insoluble fraction was discarded after centrifugation. Lyophilized soluble samples were resuspended in 250 to 500 μL of 1% NaCl followed by precipitation of AGPs with an equal volume of β-glucosyl Yariv reagent (Biosupplies, Australia) in 1% (w/v) NaCl for 16 hours at 4°C. After centrifugation at 14,000g for 1 hour, the pellet was dried and dissolved in a minimum volume of dimethyl sulfoxide, then mixed with solid sodium dithionite followed by mixing with water until the solution became a clear yellow color. The resulting solution was subjected to dialysis against 4 L of deionized water and then lyophilized.

To determine monosaccharide composition, 1 mg of either destarched dry cell wall, polysaccharide fraction, or extracted AGP was hydrolyzed by 2N trifluoracetic acid at 120°C for 2h. After the acid was evaporated, hydrolysate was re-dissolved in water and analyzed by HPAEC-PAD using CarboPac PA-20 column (Dionex, CA, USA) as described above in description of activity assay. Monosaccharide standards included L-Fuc, L-Rha, L-Ara, D-Gal, D-Glc, D-Xyl, D-Man, D-GalA and D-GlcA (all from Sigma). To determine response factors, standard curves were created using mixtures of all standard monosaccharides at different concentrations.

### RNA extraction, cDNA synthesis and Real-time qPCR

Total RNA was extracted from the leaves of 3-week-old plants using SV Total RNA Isolation kit (Promega) and cDNA synthesis was performed with the Superscript III First Strand synthesis system (Invitrogen) following the manufacturer’s recommendations. Maxima SYBR Green qPCR Master Mix (2X) (Fermentas) with appropriate primers (S1 Table) and Roche Lightcycler 480 qPCR (Plant Science Institute at Iowa State University) were used to determine relative expression of the genes. Relative expression levels were calculated in comparison to an appropriate control gene *ACTIN-2* (*At3g18780*). The 2^−ΔΔCt^ method [33] was used for determining differences between transcript copy numbers in wild type and transgenic plants.

### Growth assay for phenotypic characterization and stress tolerance determination

Measurement of hypocotyl and root length was done using ImageJ software (http://rsb.info.nih.gov/ij/) from the images of seedlings germinated and grown for 5 days in the dark on solid Murashige and Skoog Media (MS) (Sigma-Aldrich, M 5524).

To evaluate root tolerance to NaCl and mannitol stress, wild type and transgenic AnF seeds were germinated in unstressed conditions for 3 days on plates with solid MS media and then transferred to MS plates containing either 150mM NaCl or 300mM mannitol. Length of roots was measured manually after 5 days of growth under a 16 h light /8 h dark cycle at 21°C.

## Results

### Preparation of transgenic Arabidopsis plants expressing α-1,2-fucosidase in the apoplast

**Fig 1:**
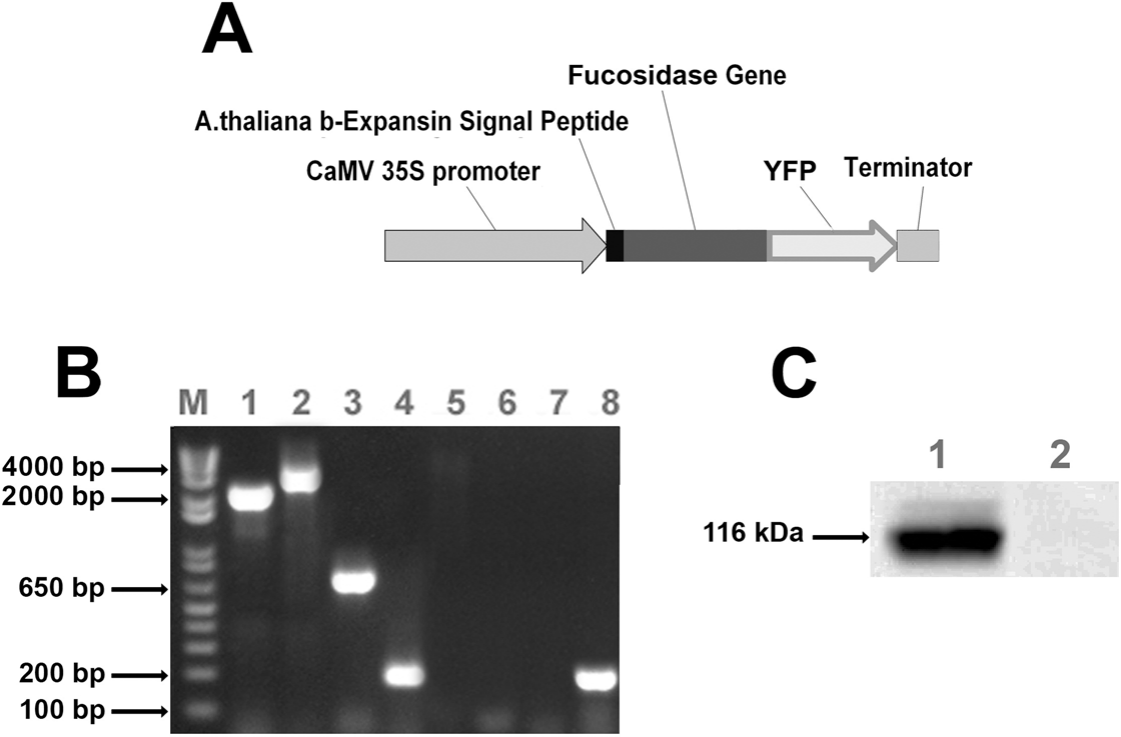
(A) The expression cassette of the vector developed for Arabidopsis transformation. Abbreviations: CaMV 35S –Tetramer of Cauliflower Mosaic virus 35S RNA Promoter, YFP, yellow fluorescent protein coding sequence. (B) PCR analysis of genomic DNA from transgenic Arabidopsis lines transformed with microbial *A*.*nidulans* α-fucosidase expression cassette and wild type plants. Herbicide resistant lines were confirmed to harbor the full construct using four pairs of primers (see S1 Table for sequences). Lane 1—amplification of *AnF* genes from corresponding transgenic lines; Lane 5—amplification of the same genes from *Col*-0 wild type plant; Lane 2—amplification of hybrid fragment containing *AnF* gene linked to the *YFP* from mutant lines; Lane 6—amplification of the *AnF*-*YFP* fragment from *Col*-0 wild type plant; Lane 3—amplification of *YFP* gene from the AnF line; Lane 7 –amplification of *YFP* from *Col-0* wild type plant; Lane 4—amplification of *A*. *thaliana ACTIN*-2 gene fragment from AnF line, and Lane 8 –amplification of *ACTIN*-2 from *Col*-0 wild type plant. Analysis was done for three independent transgenic lines for each construction; picture shows results of PCR for single plant of each mutant line. (C) Western blot analysis of total proteins from apoplast of Arabidopsis AnF transgenic and wild type plants. The corresponding microbial fucosidase fused with YFP (116kDa) were found in transgenic lines and not in *Col*-0 control plants. Blots were produced using GFP monoclonal antibodies (1:5000 dilution).

The full-length cDNA of *A*.*nidulans* α-fucosidase (AN8149.2) (*AnF*) was introduced into the binary expression vector pEarleyGate 101 containing the cauliflower mosaic virus 35S promoter and YFP at 3’-end. To target the translated product to the apoplast, the 5’-end of the hydrolase cDNA was fused with coding sequence of *A*. *thaliana* expansin-B signal peptide coding sequence to direct the expressed protein to the apoplast (Fig 1A).

**Fig 2:**
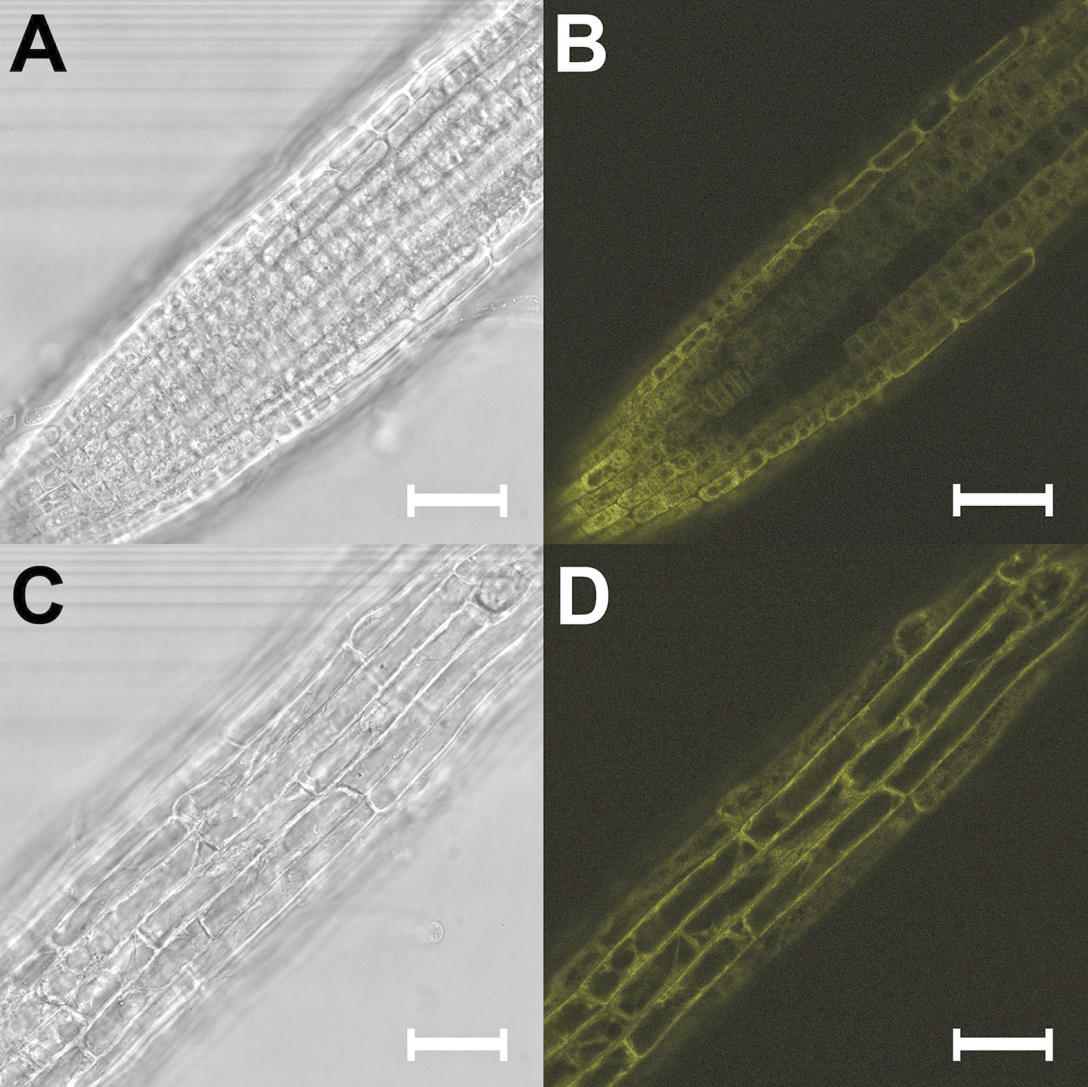
Light and confocal microscopy images of different organs of 3-week old transgenic Arabidopsis plants expressing AnF. (A) Light microscopy image of AnF expressing plant root cells. (B) Localization of AnF protein fused with YFP in the root cells. (C) Light microscopy image of AnF expressing plant stem cells. (D) Localization of AnF protein fused with YFP in the stem cells. Bars = 0.2 mm.

Three independent herbicide resistant *A*. *thaliana* transgenic plants were genotyped to confirm the presence of the complete construct (Fig 1B) introduced into the plant genomic DNA and homozygous plants were confirmed by normal segregation. The apoplastic fluids were prepared from the selected homozygous transgenic plants and analyzed by Western blot using anti-GFP antibodies. The presence of the band corresponding to the size of the α-Fucosidase protein together with YFP (116 kDa), where the predicted protein size from the sequence of *A*.*nidulans* α-fucosidase is 90 kDa and the size of YFP is 26 kDa, was observed in the transgenic plants expressing AnF, whereas no bands were observed in apoplast of wild type plants (Fig 1C). To confirm cell wall localization of the expressed AnF protein, localization of the YFP fluorescence in the transgenic plant tissues was examined by confocal microscopy (Fig 2). The fluorescence signal was observed around the cells in all organs of Arabidopsis transgenic homozygous plants. This observation, together with the results from Western blot analysis of apoplastic proteins, confirmed that AnF is localized in the Arabidopsis apoplast.

### AnF expressed in the apoplast of Arabidopsis transgenic plants maintain α-1,2-fucosidase activity

**Fig 3:**
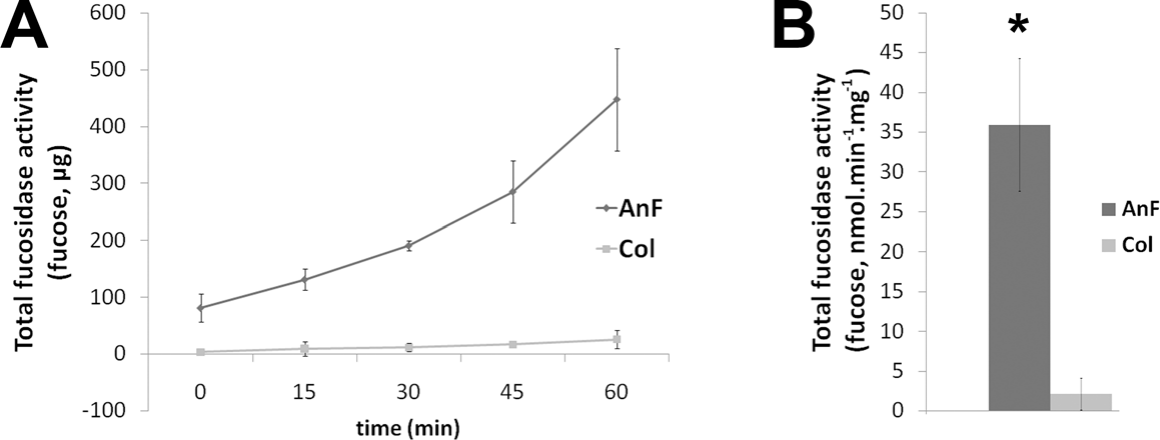
Enzymatic activity of *A*.*nidulans* fucosidase in apoplast from Arabidopsis transgenic plants assayed using 2-Fucosyllactose. Released fucose was determined by HPAEC-PAD chromatography (as described in Experimental procedures). Means represent average activity determined for three independent transgenic lines and three wild type *Col*-0 apoplasts. * Differences between transgenic lines and *Col*-0 are significant (n = 3, p<0.01). (A) Time-course of fucose released from the substrate where 1 mg of total apoplast protein was used. (B) Calculated activity of AnF in Arabidopsis apoplast.

In order to verify that the expressed AnF protein exerts its activity, an enzymatic assay was used to compare apoplastic fluids extracted from the three independent homozygous transgenic lines and three non-transformed *Col*-0 plants. 2-Fucosyllactose was used as a substrate, and the assay conditions were established using commercially available α-1,2-fucosidase. Amount of fucose released after incubation of apoplast with the substrate was measured by HPAEC as described in Material and Methods. The apoplastic fractions prepared from the transgenic plants contained significantly higher fucosidase activity in comparison with the apoplast extracted from wild type plants (Fig 3).

### Expression of AnF in Arabidopsis reduces fucosyl content in the cell wall due to defucosylation of AGP

**Fig 4:**
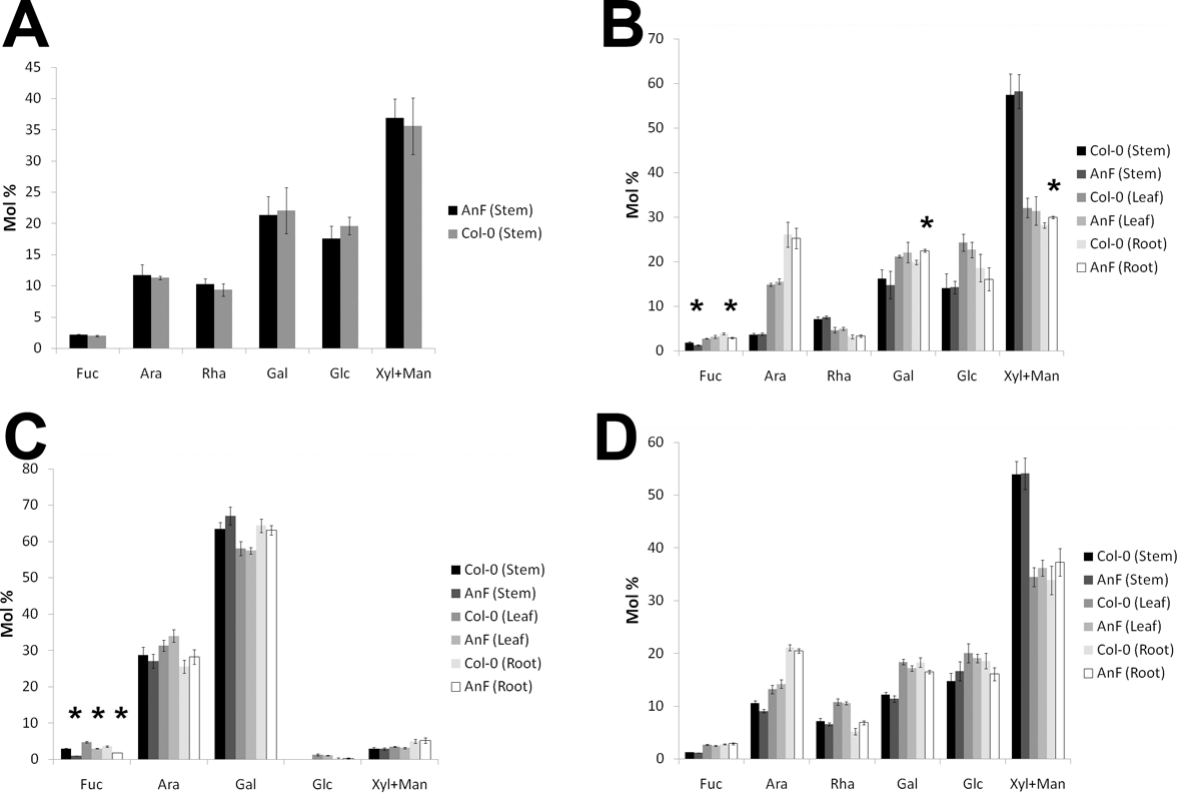
Monosaccharide composition (mol%). (A) Monosaccharide composition of total cell walls extracted from the whole 4-week-old transgenic plants expressing AnF and wild type Col-0 plants. (B) Monosaccharide composition of cell wall fractions after pectin being removed. Analysis was done using stem, leaf and root tissues of 4-week-old transgenic Arabidopsis plants expressing AnF and wild type Col-0 plants. (C) Neutral monosaccharide composition (mol%) of AGP glycan. (D) Monosaccharide composition of cell wall fraction remaining after AGP removal. Analysis was done using stem, leaf, and root tissues of 4-week-old Arabidopsis plants. * Differences between transgenic lines and *Col*-0 are significant (n = 3, p<0.05).

Compositional analysis of the cell walls prepared from wild type and AnF-expressing transgenic plants was performed to examine the effect of expressed fucosidase. Monosaccharide composition of total cell wall material prepared from whole plants did not show significant difference between transgenic and wild type plants (Fig 4A; S2A Table). However, the hemicellulose fractions from stem and root cell walls, after CDTA-soluble polysaccharides were removed, revealed 28% and 16% reduction of fucose residues, respectively, in comparison with hemicelluloses obtained from the stems and roots of wild type plants (Fig 4B; S2B Table). This indicated that introduced fucosidase, most likely, cleaves fucosyl residues from the components present in the hemicellulose fraction.

Next, we sought to investigate whether fucose was cleaved from specific cell wall components or cleaved randomly from the components remaining after CDTA treatment (AGPs, xyloglucan, and possibly RG-I). The soluble in 4M KOH fraction was prepared and treated with Xyloglucan Endoglucanase (XEG) to digest xyloglucan. Obtained oligosaccharides were analyzed by HPAEC to investigate the possible changes in xyloglucan molecules. No difference in xyloglucan fucose content between wild type and transgenic plants was observed (S1 Fig), indicating that AnF does not remove fucosyl residues from xyloglucan.

Next, AGPs were extracted from Arabidopsis roots and aerial parts using the protocol described earlier [32] and the monosaccharide composition of both the AGP fraction and the cell wall remaining after its extraction was determined. The amount of total AGPs extracted from cell walls of wild type and transgenic plants did not show significant difference (data not shown). The results from monosaccharide analysis of prepared AGP fractions (Fig 4C and 4D; S3 Table) demonstrated that reduction of the fucosyl content observed in the hemicellulose fraction from transgenic AnF plants is due to a significant decrease of fucose present in the AGP fraction (Fig 4C; S3A Table). There was no significant difference in monosaccharide composition of the cell wall fractions remaining after AGPs were removed between transgenic and wild type plants (Fig 4D; S3B Table). Since no pectin-related monosaccharides were detected in AGP fraction, it is plausible to conclude that AnF expressed in Arabidopsis apoplast defucosylates primarily AGP and not other cell wall components.

### Microbial fucosidase expressed in Arabidopsis apoplast affects expression of several fucosyltransferases

**Fig 5:**
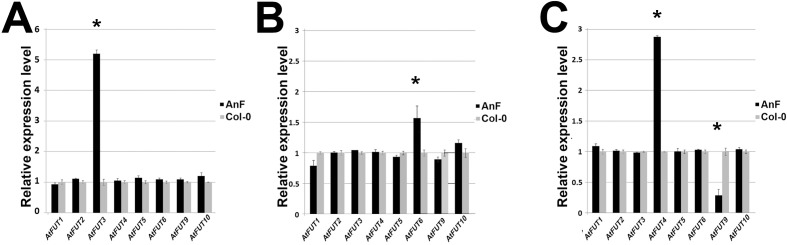
Real-time qPCR analysis of *FUT* gene expression in transgenic lines AnF and wild type plants. Relative expression levels were calculated as comparison to the *ACTIN-2* reference gene, whose expression was not affected. 2^−ΔΔCt^ method was used for determining difference between transcripts copy numbers in wild-type and transgenic plants. * Differences between transgenic lines and *Col*-0 are significant. Analysis of *AtFUT* genes expression level were done separately for: (A) Stems. (B) Leaves. (C) Roots.

It was shown before that post-synthetic modification of cell walls induces transcriptional upregulation of genes involved in the synthesis of the cell wall component being modified, most likely as a compensatory response of a plant [25]. To investigate whether the post-synthetic defucosylation of cell walls in AnF-expressing plants exerts similar effects, expression of ten *FUT* genes predicted to be involved in polysaccharide fucosylation was studied using qPCR. Obtained results demonstrated that Arabidopsis AnF plants have higher expression of *FUT3* in stems (5.2-fold), *FUT6* in leaves (1.5 fold), and *FUT4* (3 fold) in roots, whereas in roots *FUT9* is downregulated 4 fold (Fig 5).

### Reduction of fucose content in the cell wall affects root and hypocotyl elongation

**Fig 6:**
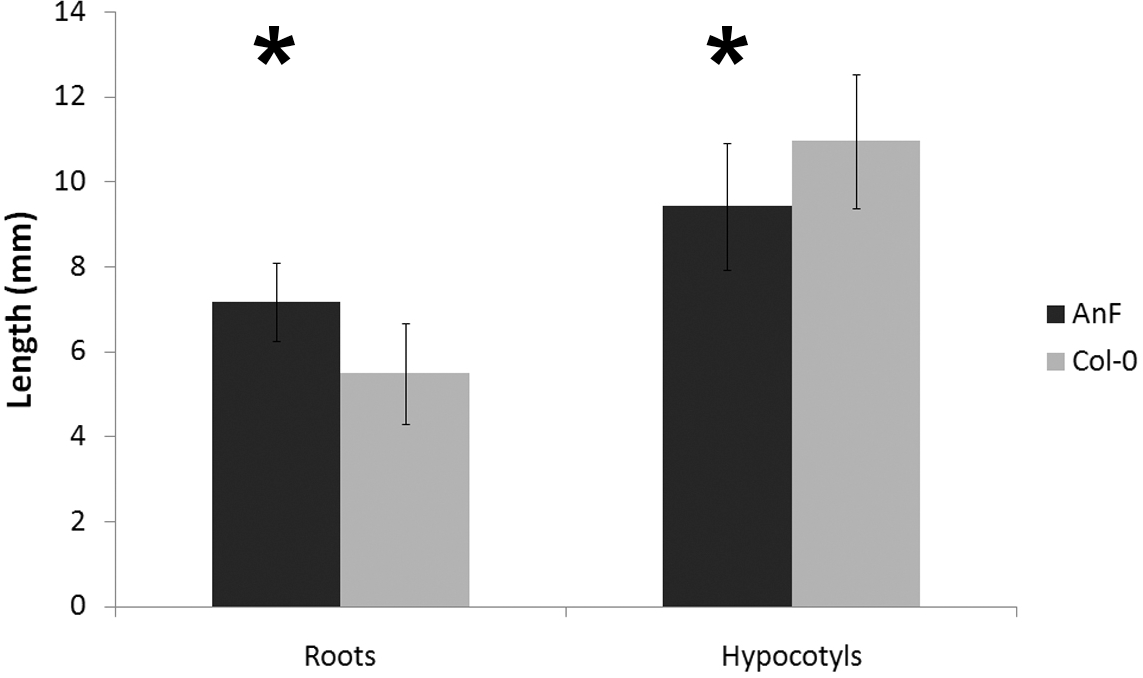
The length of primary roots and hypocotyls of AnF and wild type Arabidopsis seedlings grown in dark measured after 6 days after germination. Two independent sets of growth tests were performed where, in total, 118 AnF and 129 wild type seedlings were measured. Bars represent mean ± standard deviation of the mean. *significantly different (t-test, p<0.01).

To analyze further the plant response to different levels of cell wall de-fucosylation in different organs, the length of roots and hypocotyls of 5-day-old transgenic seedlings germinated and grown in the dark was examined. Roots of Arabidopsis AnF transgenic lines showed a 30.4% increase of length, whereas the hypocotyls were 14.1% shorter than the wild type hypocotyls grown in the same conditions (Fig 6). However, the total length of the seedlings did not show any significant difference between wild type and transgenic AnF plants–the average being 16.58 vs 16.54 mm.

## Discussion

Expression of microbial hydrolases in plants has been shown as an effective approach to introduce constitutive modifications of cell walls to reduce their recalcitrance and increase biomass digestibility [34–37]. These modifications can also induce CWI signaling which could lead to changes in plant growth [3] or plant responses towards environmental stresses [25, 38–40]. Hence, transgenic plants expressing specific microbial cell wall degrading enzymes can be used as a tool to investigate the signaling pathways induced in response to cell wall post-synthetic modifications caused by the hydrolases localized in the apoplast, which mimics the events potentially occuring during plant-microorganism interaction [25, 41].

In this study, we have shown that expression of *A*.*nidulans* α-1,2-fucosidase (AN8149.2) in the Arabidopsis apoplast causes post-synthetic defucosylation of the cell wall, primarily reducing fucosylation of AGPs, which induces differential expression of several *FUT* genes as a compensatory response and affects plant growth.

In the CAZy database [42], α-fucosidases are classified into two GH families, GH29 and GH95 [43]. It is believed that GH95 enzymes are active solely on α-1,2-linked fucose, which is present in RG-I, xyloglucan and AGP glycans [44], whereas the α-fucosidases from GH29 family recognize various types of linkages but mainly cleave 1,3/1,4-linked fucose [45]. While Fuc95A (AXY8) was shown to act specifically on xyloglucan [21], the α-1,2-fucosidase purified from *X*. *manihoti* was able to cleave fucose from AGP glycan [10]. Both proteins belong to the family GH95. The *A*.*nidulans* α-1,2-fucosidase (AnF) used in this study also belongs to family GH95, so potentially can act on RG-I, xyloglucan and AGP glycan. It was reported previously that recombinant AnF expressed in *P*.*pastoris* and tested in an *in vitro* assay was able to defucosylate xyloglucan oligomers extracted from cotton, but it was not active on p-nitrophenol (PNP)-fucoside and was not tested against AGPs [27]. The results obtained here indicate that AnF localized in the Arabidopsis apoplast cleaves fucosyl residues preferentially from AGPs and does not affect the xyloglucan molecules. It is possible that AnF is capable to cleave fucose from both glycans *in vitro*, but *in vivo*, the fucosyls in AGP are more accessible to the enzyme within the intact cell wall.

Apoplast from transgenic Arabidopsis plants expressing AnF had fucosidase activity 17-fold higher in comparison with the same type of activity in wild type apoplast. However, reduction of fucose content in transgenic total cell walls was only about 20%, and 50–70% in purified AGP fraction depending on the organ analyzed. Most likely, the enzyme concentration in the apoplast of the transgenic plants is not the limiting factor in achieving higher level of defucosylation; rather, the accessibility of fucosyls to the enzyme is different within the intact cell wall. In addition, the differential upregulation of several FUT genes observed in transgenic plants suggests the induction of a potential compensatory mechanism in response to reduction of fucosylation in their cell wall. The FUT1 protein is believed to be the only fucosyltransferase responsible for adding the terminal α-1,2-fucosyl onto xyloglucan molecules in all Arabidopsis tissues, because *fut1* mutant plants do not have any detectable fucosylated xyloglucan [46]. On the other hand, FUT4 and FUT6 were shown to fucosylate AGP. From previous reverse genetics studies, it was concluded that fucosylation of AGP in roots depend on the presence of both proteins, whereas in leaves, the FUT4 protein is mainly responsible for AGP fucosylation because FUT6 is not highly expressed [19–20]. Currently, we do not have a good explanation for why we observed upregulation of FUT6 but not of FUT4 in response to defucosylation of AGPs in leaves. Perhaps when the plant cell in leaves of AnF plants responds to post-synthetic defucosylation of AGP, this response proceeds via upregulation of the second gene (FUT6) involved in AGP fucosylation, which is still expressed in wild type leaves though at a lower level. In AnF plants, FUT4 functions with its normal capacity and may be unresponsive to post-synthetic modification of AGP in transgenic plants. There is no information available so far about FUTs involved in AGP fucosylation in Arabidopsis stems, nor the function of seven other fucosyltransferases, FUT2, FUT3, FUT5, and FUT7-FUT10. Some of those enzymes are, most likely, responsible for adding fucosyl residues to RG-I and RG-II molecules. Because the correlation between reduction of AGP fucosylation and upregulation of *FUT* genes involved in AGP fucosylation was observed in the roots and leaves of transgenic AnF plants, it is plausible to propose that *FUT3* strongly upregulated in stems of transgenic plants is also involved in fucosyltaion of AGPs specifically in stem tissues. In a future study, T-DNA mutants will be used to confirm this. The results obtained here suggest that post-synthetic cell wall defucosylation induces a compensatory mechanism that involves transcriptional regulation of the genes involved in biosynthetic processes. Perhaps, the transgenic plants try to maintain the integrity of their cell walls by synthesizing more fucosylated AGPs. This might explain why the complete cell wall defucosylation was not achieved in spite of high fucosidase activity built up in the apoplast. Similar effects of post-synthetic cell wall modification was observed earlier [25], when deacetylation of xylan or pectin in Arabidopsis induced upregulation of the RWA genes predicted to be involved in polysaccharide acetylation [47]. Such potential connection between the cell wall modification introduced post-synthetically and the transcriptional regulation of the genes involved in biosynthesis of the molecules being modified is an indication of CWI control involvement in plant compensatory mechanisms. Thus, the utilization of transgenic plants with post-synthetically modified cell walls can be a promising approach to reveal new yet uncharacterized genes involved in cell wall biosynthesis.

The Arabidopsis *mur1* plants, which are defective in producing GDP-Fuc from GDP-Man [48], have a 40% reduction of fucose present in root cell walls [49] and decreased rate of root elongation [16]. Thus, it was proposed that fucosylation of AGPs is required for normal root cell elongation [16]. However, two recent independent studies, which used Arabidopsis T-DNA mutants *fut4*, *fut6*, and *fut4*/*fut6*, demonstrated that the lack of fucose in AGP glycan does not impact root growth in normal conditions but increases their salt susceptibility [19–20]. The results obtained in our study conform to the notion that AGP fucosylation is not critical for cell elongation. The differences in root and stem length observed between AnF transgenic plants with reduced fucosylation of AGPs and wild type plants most likely is an effect of post-synthetic cell wall remodeling occurred in transgenic plants after defucosylation of AGPs. This will need more detailed investigation in the future. Roots of AnF-expressing plants did not show any significant changes in their susceptibility to salt or osmotic stress (S2 Fig), despite the fact that they possess a 50% reduction in AGP fucosylation. It is possible that the remaining 50% of AGP fucosylation is enough to support the strength of the cell wall against high salt concentration. Another explanation might be that the incorporation of the non-fucosylated AGP molecules into the cell wall affects the cell wall structure differently in comparison with defucosylation of AGPs within cell wall, which results in different cell wall resistance to salt.

## Conclusion

We demonstrated that expression of *A*.*nidulans* α-1,2-fucosidase in the Arabidopsis apoplast modifies AGP glycan without affecting other fucosylated cell wall constituents. Post-synthetic defucosylation of AGP glycan occurring within cell walls causes less impact on cell wall structural integrity in comparison with incorporation of non-fucosylated AGPs synthesized in knock-out mutant plants. This post-synthetic modification of cell wall AGPs induces differential expression of fucosyltransferases responsible for AGP fucosylation, as a possible plant compensatory response. This approach may assist in characterization of new microbial hydrolase specificities directly on the intact cell wall and also in revealing currently uncharacterized genes predicted to be involved in the synthesis of cell wall components.

## Supporting Information

S1 Fig Relative proportion of xyloglucan subunits. (DOCX)

S2 Fig Root length measurements. (DOCX)

S1 Table List of primers used in this work (5’– 3’). (DOCX)

S2 Table Neutral monosaccharide composition (mol%) of cell walls from transgenic plants expressing AnF and wild type Col-0 plants. (DOCX)

S3 Table Neutral monosaccharide composition (mol%) of AGP glycan and remainder of cell walls. (DOCX)
