# Supplementary material for: Post-Synthetic Defucosylation of AGP by Aspergillus nidulans α-1,2-Fucosidase Expressed in Arabidopsis Apoplast Induces Compensatory Upregulation of α-1,2-Fucosyltransferases
Source: PLoS One. 2016 Jul 22;11(7):e0159757. doi: 10.1371/journal.pone.0159757 (PMC4957772; doi:10.1371/journal.pone.0159757)
Supplement: S1 Fig — (DOCX) [file pone.0159757.s001.docx]

**S1 Figure. Relative proportion of xyloglucan subunits.**

Relative proportion of xyloglucan subunits generated from wild type (Col-0) and transgenic plants (AnF) after digestion of the 4M KOH hemicellulose fraction extracted from cell wall of a whole 4-week-old seedling with xyloglucan endoglucanase (XEG) and analyzed by high-performance anion exchange chromatography (HPAEC). Identification of the peaks was performed by matrix-assisted laser desorption/ionization-time of flight (MALDI-TOF).
