# Supplementary material for: Post-Synthetic Defucosylation of AGP by Aspergillus nidulans α-1,2-Fucosidase Expressed in Arabidopsis Apoplast Induces Compensatory Upregulation of α-1,2-Fucosyltransferases
Source: PLoS One. 2016 Jul 22;11(7):e0159757. doi: 10.1371/journal.pone.0159757 (PMC4957772; doi:10.1371/journal.pone.0159757)
Supplement: S2 Fig — (DOCX) [file pone.0159757.s002.docx]

**S2 Figure. Root length measurements.**

Root length measurements of wild type (Col-0) and transgenic plants (AnF) after growth in normal and stressed condtions.

(A) root length measurements of osmotically stressed plants grown for 5 days on MS media + 300 mM mannitol.

(B) root length measurements of salt stressed plants grown for 5 days on MS media + 150 mM NaCl. n=20.
