# Supplementary material for: Post-Synthetic Defucosylation of AGP by Aspergillus nidulans α-1,2-Fucosidase Expressed in Arabidopsis Apoplast Induces Compensatory Upregulation of α-1,2-Fucosyltransferases
Source: PLoS One. 2016 Jul 22;11(7):e0159757. doi: 10.1371/journal.pone.0159757 (PMC4957772; doi:10.1371/journal.pone.0159757)
Supplement: S1 Table — (DOCX) [file pone.0159757.s003.docx]

**S1 Table.** **List and sequences of primers used in this work.**

5’ – 3’

AnF-Forw: TCTAGAATGCGGAAGACCACTCTGTT

AnF-Rev: GGTACCTCACCATTTACGAACGATA

AtSP-Forw: CCATGGATGGGTCATCTTGGGTTCTT

AtSP-Rev: TCTAGAACCGTAGCCGCTCACAGAAG

Actin_Forw: GAAACCCTCGTAGATTGGCA

Actin_Rev: CTCTCCCGCTATGTATGTCGC

YFP-F: GGATCCCATATGAGGGTGAGCAAGGGCGAGGA

YFP-R: TCTAGACTTGTACAGCTCGTCCATGC

AtFUT1-F: CCTGAAGTAGACACACTAGTGG

AtFUT1-R: GAGCTTTGCCATTATGCATC

AtFUT2-F: TGAGAAATCCGGCGTATCTCC

AtFUT2-R: GGTGACCAAGACAGATTTGAG

AtFUT3-F: CTTCAAGCCAAGTTCTGAACCG

AtFUT3-R: GCCCATGCTCTCTTACTTTCC

AtFUT4-F: TCACCACTTGGGTCGGTATCT

AtFUT4-R: CTTGTGTAGCCAATTCAGGC

AtFUT5-F: TACTACCACGCTCATTTGTCG

AtFUT5-R: GCCAGAGAACTCTGGATAGAG

AtFUT6-F: GTTGCTCATAGTCTTGGAGG

AtFUT6-R: AAGACCATCGTTCCCACGATC

AtFUT9-F: CAGACAAGAAGCTTCACGACC

AtFUT9-R: TCCATCGACATGGCCCTAAC

AtFUT10-F: CCAATGCAGACGAGAGACTTG

AtFUT10-R: GTACTCTGGATAGAGAGATGC
