# Supplementary material for: Post-Synthetic Defucosylation of AGP by Aspergillus nidulans α-1,2-Fucosidase Expressed in Arabidopsis Apoplast Induces Compensatory Upregulation of α-1,2-Fucosyltransferases
Source: PLoS One. 2016 Jul 22;11(7):e0159757. doi: 10.1371/journal.pone.0159757 (PMC4957772; doi:10.1371/journal.pone.0159757)
Supplement: S2 Table — (DOCX) [file pone.0159757.s004.docx]

**S2** **Table**. **Neutral monosaccharide composition (mol%) of cell walls from transgenic plants expressing AnF and wild type Col-0 plants.**

(A) Monosaccharide composition of total cell walls extracted from the whole 4-week-old plants.

(B) Monosaccharide composition of cell wall fractions after pectin being removed. Analysis was done using stem, leaf and root tissues of 4-week-old Arabidopsis plants.

| **A** |  | |  | | | | |  | | | |  | | | | |  | | | |  | | | | |  |  |  |  |
| --- | --- | --- | --- | --- | --- | --- | --- | --- | --- | --- | --- | --- | --- | --- | --- | --- | --- | --- | --- | --- | --- | --- | --- | --- | --- | --- | --- | --- | --- |
|  | | | Fuc | | | Ara | | | Rha | | | | Gal | | | | | Glc | | | | Xyl+Man | | | | |  |  |  |
| Col-0 | | | 2.0±0.1 | | | 11.3±0.3 | | | 9.4±1.0 | | | | 22.1±3.7 | | | | | 19.6±1.4 | | | | 35.6±4.5 | | | | |  |  |  |
| AnF | | | 2.2±0.1 | | | 11.7±1.7 | | | 10.3±0.9 | | | | 21.4±2.9 | | | | | 17.6±2.0 | | | | 36.9±3.1 | | | | |  |  |  |
| **B** |  | | |  | | | | | | |  | | | | |  | | | |  | | | | |  | | | | |
|  |  | Fuc | | | Ara | | | | | Rha | | | | Gal | | | | | Glc | | | | | Xyl+Man | | | |  |  |
| Col-0 (Stem) | | 1.9±0.2 | | | | | 3.7±0.3 | | | 7.1±0.6 | | | | | 16.2±2.0 | | | | 14.1±3.2 | | | | 57.4±4.8 | | | | | |  |
| AnF (Stem) | | ***1.3±0.1** | | | | | 3.8±0.3 | | | 7.6±0.3 | | | | | 14.8±3.1 | | | | 14.3±1.4 | | | | 58.2±3.8 | | | | | |  |
| Col-0 (Leaf) | | 2.8±0.1 | | | | | 14.9±0.3 | | | 4.7±0.6 | | | | | 21.2±0.3 | | | | 24.3±1.9 | | | | 32.1±2.2 | | | | | |  |
| AnF (Leaf) | | 3.2±0.3 | | | | | 15.6±0.6 | | | 5.0±0.3 | | | | | 22.1±2.3 | | | | 22.7±1.7 | | | | 31.4±3.2 | | | | | |  |
| Col-0 (Root) | | 3.9±0.2 | | | | | 26.1±2.8 | | | 3.2±0.4 | | | | | 19.9±0.5 | | | | 18.6±3.1 | | | | 28.2±0.6 | | | | | |  |
| AnF (Root) | | ***2.9±0.2** | | | | | 25.3±2.3 | | | 3.4±0.3 | | | | | ***22.5±0.3** | | | | 16.1±2.6 | | | | ***30.0±0.3** | | | | | |  |

*significantly different (t-test, p<0.01, n=3)
