# Supplementary material for: Post-Synthetic Defucosylation of AGP by Aspergillus nidulans α-1,2-Fucosidase Expressed in Arabidopsis Apoplast Induces Compensatory Upregulation of α-1,2-Fucosyltransferases
Source: PLoS One. 2016 Jul 22;11(7):e0159757. doi: 10.1371/journal.pone.0159757 (PMC4957772; doi:10.1371/journal.pone.0159757)
Supplement: S3 Table — (DOCX) [file pone.0159757.s005.docx]

**S3 Table. Neutral monosaccharide composition (mol%) of AGP glycan and remainder of cell walls.**

(A) Monosaccharide composition of AGP.

(B) Monosaccharide composition of cell wall fraction remaining after AGP removal. Analysis was done using stem, leaf, and root tissues of 4-week-old Arabidopsis plants.

**A**

|  | Fuc | Ara | Gal | Glc | Xyl+Man |
| --- | --- | --- | --- | --- | --- |
| Col-0 (Stem) | ***3.0±0.1** | 28.8±2.1 | 63.5±1.8 | N.D | 3.0±0.4 |
| AnF (Stem) | ***1.0±0.0** | 27.1±1.9 | 67.1±2.5 | N.D | 2.9±0.3 |
| Col-0 (Leaf) | ***4.8±0.2** | 31.3±1.5 | 58.1±1.9 | 1.3±0.3 | 3.5±0.2 |
| AnF (Leaf) | ***3.0±0.1** | 34.0±1.7 | 57.5±0.9 | 1.1±0.1 | 3.2±0.2 |
| Col-0 (Root) | ***3.6±0.2** | 25.5±1.8 | 64.4±1.9 | 0.2±0.2 | 5.0±0.6 |
| AnF (Root) | ***1.8±0.0** | 28.2±2.0 | 63.1±1.3 | 0.3±0.1 | 5.2±0.8 |

**B**

|  | Fuc | Ara | Rha | Gal | Glc | Xyl+Man |
| --- | --- | --- | --- | --- | --- | --- |
| Col-0 (Stem) | 1.3±0.0 | 10.6±0.5 | 7.2±0.5 | 12.2±0.5 | 14.8±1.5 | 53.9±2.5 |
| AnF (Stem) | 1.2±0.0 | 9.1±0.3 | 6.6±0.3 | 11.4±0.6 | 16.7±1.8 | 54.1±3.0 |
| Col-0 (Leaf) | 2.7±0.1 | 13.2±0.8 | 10.8±0.6 | 18.4±0.5 | 20.1±1.8 | 34.5±1.8 |
| AnF (Leaf) | 2.5±0.1 | 14.2±0.8 | 10.6±0.3 | 17.2±0.5 | 19.1±0.8 | 36.2±1.5 |
| Col-0 (Root) | 2.8±0.1 | 21.1±0.6 | 5.2±0.6 | 18.3±0.9 | 18.6±1.5 | 33.9±2.7 |
| AnF (Root) | 2.9±0.2 | 20.5±0.4 | 6.9±0.4 | 16.5±0.3 | 16.1±1.2 | 37.3±2.6 |
